# MyD88 Contributes to TLR3-Mediated NF-κB Activation and Cytokine Production in Macrophages

**DOI:** 10.3390/cells14191507

**Published:** 2025-09-27

**Authors:** Zhuodong Chai, Yuqi Zhou, Ling Yang, Yan Zhang, Sukria Hossain, Sahelosadat Hajimirzaei, Jiaqian Qi, Guoying Zhang, Yinan Wei, Zhenyu Li

**Affiliations:** Department of Pharmaceutical Sciences, Irma Lerma Rangel School of Pharmacy, Texas A&M University, College Station, TX 77845, USA; zdmaccc@tamu.edu (Z.C.); yuqi.zhou@tamu.edu (Y.Z.); ling.yang@tamu.edu (L.Y.); yzh422@tamu.edu (Y.Z.); sukriahossain78@gmail.com (S.H.); sahelmirzaei@tamu.edu (S.H.); qijq@tamu.edu (J.Q.); gzh223@tamu.edu (G.Z.); yinan.wei@tamu.edu (Y.W.)

**Keywords:** macrophage, TLR3, NF-KB, MyD88, TRIF, cytokine, TNF, IL-6, inflammation

## Abstract

Toll-like receptor 3 (TLR3) initiates antiviral and inflammatory responses exclusively through the adaptor protein TRIF (TIR-domain-containing adapter-inducing interferon-β). In contrast, MyD88 (myeloid differentiation primary response 88), a central adaptor for most other TLRs, is traditionally considered dispensable for TLR3 signaling. Here, we demonstrate that MyD88 directly contributes to TLR3-mediated NF-κB activation and cytokine production in macrophages. Bone marrow-derived macrophages (BMDMs) from MyD88 deficient mice exhibited significantly attenuated NF-κB activation in response to the TLR3 agonist polyinosinic–polycytidylic acid (poly(I:C)) compared to wild-type cells, as evidenced by the reduced phosphorylation of NF-κB p65 and IκBα, as well as IκBα degradation. Consistently, pro-inflammatory cytokine production, including IL-6, TNF-α, and IFN-β, was attenuated in MyD88-deficient BMDMs in vitro following stimulation by poly(I:C) or poly(A:U), another TLR3 agonist. Blood concentrations of IL-6, TNF-α, and IFN-β were significantly reduced in both TRIF-deficient mice and MyD88-deficient mice challenged by the i.p. injection of poly(I:C). Mechanistic analyses revealed that MyD88 physically associates with activated TLR3 upon poly(I:C) stimulation, and that TLR3 engagement triggered MyD88 oligomerization, which was absent in TLR3 or TRIF deficient macrophages. Our findings highlight a previously unrecognized dual-adaptor mechanism for TLR3, wherein MyD88 recruitment amplifies NF-κB signaling dynamics by bridging TLR3 to the canonical NF-κB activation cascade and robust cytokine induction. This study expands the paradigm of TLR3 signaling by establishing MyD88 as a direct contributor to TLR3-driven innate immune responses, offering new insight into cross-talk between MyD88-dependent and -independent pathways.

## 1. Introduction

Toll-like receptors (TLRs) are a family of pattern recognition receptors (PRRs) that recognize a variety of pathogen-associated molecular patterns (PAMPs), including bacterial lipopolysaccharides (LPSs), flagellin, viral RNA, and unmethylated CpG DNA [1,2,3,4,5]. Upon recognizing their specific ligands, TLRs trigger intracellular signaling cascades that lead to the production of pro-inflammatory cytokines, type I interferons (IFN-I), and other mediators essential for mounting an effective immune response, thus playing a critical role in innate immunity. The TLR family comprises 10 members in humans (TLR1–10) and 13 in mice (TLR1–13), although TLR10 in mice is non-functional [6,7]. TLRs are expressed either on the cell surface or within endosomal compartments, reflecting their specialized roles in detecting extracellular or intracellular pathogens. For instance, TLR4 recognizes lipopolysaccharides (LPSs) on the Gram-negative bacterial outer membrane and TLR5 responds to flagellin on the bacteria surface, whereas TLR3 detects double-stranded RNA (dsRNA) in the endosome, a molecular feature characteristic of many viruses [8,9].

A key feature of TLR signaling is its reliance on adaptor proteins, which mediate the downstream activation of signaling pathways. Among the TLR family members, most are known to depend on the adaptor protein Myeloid differentiation primary response protein 88 (MyD88) for downstream signaling [7,10,11,12]. MyD88-dependent signaling is essential for rapid immune responses to many bacterial and viral infections. It promotes the activation of nuclear factor kappa-light-chain-enhancer of activated B cells (NF-κB) and mitogen-activated protein kinases (MAPKs), driving the expression of pro-inflammatory cytokines such as interleukin-10 (IL-10), interleukin-18 (IL-18), and tumor necrosis factor-alpha (TNF-α) [2,13,14]. TLR2 and TLR4 are exceptions, as they can also utilize a second adaptor protein, TIR-domain-containing adapter-inducing interferon-β (TRIF), enabling it to elicit both MyD88-dependent and MyD88-independent responses [15,16].

TLR3 is unique among the TLR family as it relies exclusively on TRIF for downstream signaling. In 2001, TLR3 was identified as a receptor for double-stranded RNA (dsRNA) and was shown to mediate MyD88-independent signaling via TRIF [17,18,19]. Upon recognizing dsRNA, TLR3 initiates TRIF-mediated activation of NF-κB and interferon regulatory factor 3 (IRF3), leading to the production of pro-inflammatory cytokines and type I interferons, respectively. This TRIF-dependent pathway is critical for antiviral immunity and is considered a defining feature that distinguishes TLR3 from other MyD88-dependent TLRs [20,21,22].

Although the canonical model posits that TLR3 signals exclusively via TRIF, the potential involvement of MyD88 in TLR3 signaling has remained an intriguing and contentious question. Initial observations by Alexopoulou et al. actually suggested that MyD88 contributes to TLR signaling [19]. They reported that MyD88-deficient dendritic cells and macrophages showed impaired production of IL-12, IL-6 and nitric oxide in response to poly(I:C). However, their findings were largely set aside after the discovery of TRIF, as later studies found no defect in poly(I:C)-induced cytokines in MyD88^-/-^ cells under their experimental conditions [17,23]. Nevertheless, a number of reports over the past decade revealed that MyD88 can modulate TLR3 pathways in specific contexts. MyD88 can either positively or negatively regulate TLR3 signaling [17,19,23,24,25,26].

Macrophages are pivotal mediators of innate immunity. Here, we show that MyD88 can substantially contribute to TLR3-driven NF-κB activation and cytokine production in macrophages. Specifically, in primary mouse macrophages stimulated with poly(I:C), loss of MyD88 causes a discernible reduction in IκBα degradation and NF-κB p65 phosphorylation, along with blunted transcription and secretion of IL-6 and IFN-β, compared to wild-type cells. These effects, while not as complete as those observed in TRIF-deficient macrophages, establish that MyD88 is required for optimal NF-κB signaling downstream of TLR3. Our data suggest that the canonical TRIF-dependent pathway of TLR3 is complemented by a MyD88-dependent arm that amplifies NF-κB activation and fine-tunes cytokine outputs. These insights challenge the long-held view of TLR3 as a purely MyD88-independent receptor and reveal a new layer of complexity in TLR signal transduction.

## 2. Materials and Methods

### 2.1. Mice

Wild-type C57BL/6J (JAX: 000664), Tlr3^-/-^ (JAX: 005217), Trif^-/-^ (JAX: 005037), and Myd88^-/-^ (JAX: 009088) were obtained from the Jackson Laboratory. All animals were housed at the Texas A&M University Animal Care Facility under standard conditions, following guidelines set forth by the National Institutes of Health and the Institutional Animal Care and Use Committee. All experiments were approved by the Institutional Animal Care and Use Committee, and adult mice aged 8 to 12 weeks were used for this study.

### 2.2. Bone Marrow Derived Macrophages (BMDMs) Culture

BMDMs were obtained following established protocols [5] and seeded into either 12-well or 96-well cell culture plates at a density of 1 × 10^6^ cells per well in 1 mL of RPMI-1640 medium supplemented with 15% L929-cell conditioned medium (LCM). After allowing the cells to adhere overnight, the culture medium was replaced with Opti-MEM (Life Technologies, Carlsbad, CA, USA, Cat#31985-070) prior to treatment. Specific concentrations of poly(I:C) or poly(A:U) were added or transfected by Lipofectamine 3000, with specific concentrations and incubation times indicated in the corresponding figures. After incubation, the supernatant was collected for the cytotoxicity or ELISA analysis, and the cell pellet was harvested for Western blot assay. Poly(A:U) was purchased from InvivoGen, San Diego, CA, USA (catalog no. tlrl-pau), and poly(I:C) (LMW) was purchased from InvivoGen, San Diego, CA, USA (catalog no. tlrl-picw). According to the manufacturer, both reagents were validated using TLR2- and TLR4-reporter cell assays to confirm the absence of contaminants such as lipoproteins and endotoxins.

### 2.3. ELISA Analysis

The levels of IL-6, IL-1β, and TNF-α in the cell culture medium or serum were quantified using Invitrogen ELISA kits (Thermo Fisher Scientific, Waltham, MA, USA) following the protocols provided by the manufacturer.

### 2.4. Cytotoxicity

Cell death was assessed using the CytoTox 96 Non-Radioactive Cytotoxicity Assay Kit (Promega, Madison, WI, USA, Cat#G1780), which measures lactate dehydrogenase (LDH) release. The assay was performed according to the manufacturer’s instructions.

### 2.5. Western Blotting

NFκB pathway activation and the basal protein expression levels were evaluated by Western blot analysis. Cell lysates were probed with specific antibodies targeting NFκB p65, p-p65, IκB, p-IκB, TLR3, MyD88, TRIF, Flag and actin. For NF-κB signaling analysis, NF-κB p65, phosphorylated p65 (p-p65), IκB, and phosphorylated IκB (p-IκB) were detected using specific antibodies from Cell Signaling Technology, Danvers, MA, USA (CST; Cat# 8242, 3031, 4814, and 2859) at a 1:1000 dilution. TLR3, MyD88, and TRIF were probed using specific antibodies at a 1:1000 dilution: TLR3 (Novus Biologicals, Centennial, CO, USA, Cat# NBP2-24875), MyD88 (CST, Cat# 4283), and TRIF (Novus Biologicals, Cat# NB120-13810SS). Flag-tagged proteins were recognized using an anti-Flag antibody (Sigma-Aldrich, St. Louis, MO, USA, F1804) at a 1:1000 dilution. Actin served as a loading control and was detected with anti-actin (CST, Cat#4970) at 1:1000 dilution. All primary antibodies were diluted at 1:1000 in TBS containing 5% BSA. The antibodies used in this study are summarized in Table 1. After overnight incubation with primary antibodies at 4 °C, membranes were washed three times with TBST (20 mM Tris-HCl, 150 mM NaCl, 0.1% Tween-20) and then incubated for 2 h at room temperature with the appropriate HRP-conjugated secondary antibodies. Goat anti-mouse or anti-rabbit secondary antibodies conjugated with horseradish peroxidase (HRP) were purchased from Jackson ImmunoResearch Laboratories, West Grove, PA, USA (Cat# 115-035-003, 111-035-003) and diluted at 1:3000 and 1:15,000, respectively, in TBS containing 5% BSA.

All Western blot were imaged using the ChemiDoc MP Imaging System (Bio-Rad Laboratories, Hercules, CA, USA)or Azure Imaging Systems (Azure Biosystems, Dublin, CA, USA).

### 2.6. RNA Extraction and RT-qPCR

Total RNA was extracted from cell samples using Trizol reagent (Life Technologies, Gaithersburg, MD) following the manufacturer’s protocol. Briefly, cells were lysed in Trizol reagent, and the lysates were mixed with chloroform. After 5 min at room temperature, the homogenates were centrifuged at 12,000× *g* for 15 min at 4 °C. The aqueous phase was carefully transferred to a new tube, mixed with an equal volume of isopropanol, and incubated for 10 min. RNA was pelleted by centrifugation at 12,000× *g* for 8 min at 4 °C. The resulting pellet was washed with 500 µL of 70% ethanol, followed by centrifugation at 12,000× *g* for 5 min at 4 °C. After removing the supernatant, the RNA pellet was air-dried and dissolved in sterile water. RNA concentration and purity were determined using a NanoDrop spectrophotometer (Thermo-Fisher Scientific, MA, USA).

To eliminate potential DNA contamination, total RNA was treated with DNase I (Thermo-Fisher Scientific, MA, USA) according to the manufacturer’s instructions. Subsequently, 1 µg of DNase-treated RNA was reverse-transcribed into cDNA using the RevertAid First Strand cDNA Synthesis Kit (Thermo-Fisher Scientific, MA, USA) following the provided protocol.

Quantitative PCR (qPCR) was performed to assess the expression levels of IL-6, TNF-α, and IFN-β mRNA. Reactions were carried out in triplicate using specific primers for each target gene and a reference gene RPLP0. qPCR assays were conducted on 384-well plates using a Roche LightCycler 480 instrument (Roche, Basel, Switzerland). The relative expression levels of cytokine genes were calculated using the 2^−ΔΔCT^ method.

### 2.7. Plasmid Construction and Transient Expression of TLR3 in CHO Cells

The full-length human TLR3 coding sequence was obtained from Sino Biological, Beijing, China (Cat# MG50161-CF) and cloned into the pCMV3 expression vector between the KpnI and NotI restriction sites to create pCMV3-TLR3-Flag. The vector includes a C-terminal Flag epitope, which facilitates detection of TLR3 protein. All sequences was verified by sequencing.

For transient expression of TLR3 in CHO cells, cells were seeded in 6-well plates at 70–80% confluency and transfected with plasmid pCMV3-TLR3-Flag using Lipofectamine 3000 (Thermo-Fisher Scientific, Waltham, MA, USA) according to the manufacturer’s instructions. Briefly, 5 µg of plasmid DNA and 5 µL of Lipofectamine 3000 were diluted in Opti-MEM medium (Gibco, Thermo Fisher Scientific, Waltham, MA, USA) in separate tubes, mixed gently, and incubated for 5 min at room temperature. The DNA-Lipofectamine mixture was then combined and incubated for an additional 20 min before being added dropwise to CHO cells. Cells were first incubated for 6–8 h in Opti-MEM medium containing the DNA-Lipofectamine 3000 transfection complex. After this incubation, the transfection medium was replaced with complete medium (DMEM supplemented with 10% fetal bovine serum and 1% penicillin-streptomycin), and the cells were cultured for an additional 48 h before being harvested for downstream analyses.

### 2.8. Co-Immunoprecipitation

Total protein lysates were prepared from CHO cells transiently expressing TLR3-Flag using an IP lysis buffer containing 1% NP-40, 50 mM Tris (pH 7.5), and 150 mM NaCl. Protein concentration was quantified, and 1 mg of total protein from each sample was incubated with 2 μL of anti-Flag antibody or 2 μL of anti-MyD88 antibody. The samples were gently rotated at 4 °C overnight to allow the formation of immune complexes. After incubation, protein A/G agarose beads (Blossom Biosciences, Santa Clara, CA, USA) were added to the samples, and the mixtures were rotated for an additional 4 h at 4 °C. The beads with bound immune complexes were washed three times with a wash buffer consisting of 1% NP-40, 50 mM Tris–HCl (pH 7.5), and 150 mM NaCl to remove nonspecifically bound proteins. The precipitated proteins were eluted by boiling in 2% SDS protein loading buffer. The eluted proteins were separated by SDS-PAGE and analyzed by immunoblotting with appropriate antibodies. This method was used to assess the interaction between TLR3 and MyD88 in the transient expression system.

### 2.9. MyD88 Oligomerization by Crosslinking

BMDMs were seeded into 12-well plates and cultured overnight. The cells were stimulated with 1 μg/mL poly(I:C) for the indicated time periods. After stimulation, cells were washed once with PBS and resuspended in caspase buffer containing PI8340 protease inhibitor (composition: 10 mM PIPES, pH 7.2, 10% (*w*/*v*) sucrose, 100 mM NaCl, and 0.1% (*w*/*v*) CHAPS hydrate). Cells were subjected to three cycles of freeze–thaw using dry ice and a 37 °C water bath. The lysates were first centrifuged at 500× *g* for 5 min at 4 °C to remove large cell debris, followed by a second centrifugation at 15,000× *g* for 10 min at 4 °C to clarify the supernatant. The resulting supernatant was then incubated with freshly prepared 2 mM disuccinimidyl suberate (DSS) at room temperature for 2 h with constant rotation to crosslink MyD88 oligomers. After crosslinking, samples were centrifuged again at 15,000× *g* for 10 min at 4 °C. Approximately 200 μL of the supernatant was collected, mixed with SDS loading buffer containing 5% β-ME, and boiled for 5 min before being subjected to SDS-PAGE and Western blot analysis.

### 2.10. Quantification and Statistical Analysis

Statistical analyses were conducted using Prism GraphPad version 10.0.0. Data are presented as mean ± SD. For comparisons between two groups with normally distributed data and equal variance, a two-sided Student’s t-test was applied. For multi-group comparisons, one-way ANOVA followed by Holm–Sidak multiple comparison tests was used. Time-course studies were analyzed using two-way repeated measures ANOVA with Holm–Sidak multiple comparison tests. A *p*-value of less than 0.05 was considered statistically significant.

## 3. Results

### 3.1. NF-κB Activation Is Significantly Reduced in MyD88^-/-^ Cells Treated with Poly(I:C)

**Figure 1:**
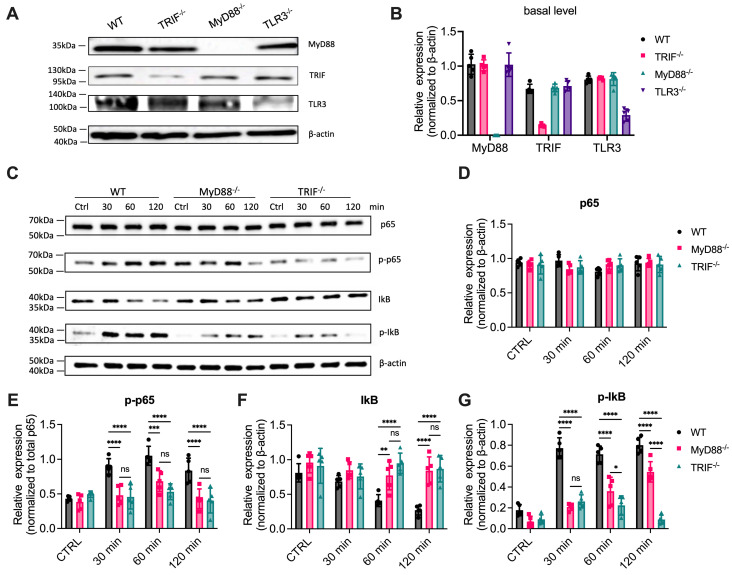
MyD88 contributes to NF-κB activation in response to poly(I:C). (**A**) Western blot analysis of MyD88, TRIF, and TLR3 basal expression levels in unstimulated cells. β-actin was used as a loading control. (**B**) Quantification of basal protein expression levels shown in (**A**). Data represent the mean ± SEM of three independent experiments. Data are presented as mean ± SD (N = 3, biological replicates). (**C**) Cells were stimulated with 1 µg/mL poly(I:C), and total protein lysates were collected at the indicated time points (0, 30, 60, and 120 min). Western blot analysis was performed to detect p65, phosphorylated p65 (p-p65), IκB, and phosphorylated IκB (p-IκB), with β-actin used as a loading control. (**D**–**G**) Quantification of protein band intensities from (**C**) was performed using ImageJ (1.54g)software. Relative intensities of (**D**) p65, (**F**) IκB, and (**G**) p-IκB were normalized to β-actin and presented as fold changes compared to controls (CTRL), while (**E**) p-p65 was normalized to the total p65 (**D**). Data are presented as mean ± SD (N = 5, biological replicates). Statistical analysis for comparisons among multiple groups was performed using two-way ANOVA followed by Sidak’s multiple comparisons testing. ns, not significant,* *p* < 0.05, ** *p* < 0.01, *** *p* < 0.005, **** *p* < 0.0001.

It has been well accepted that TLR3 relies on the adapter TRIF to activate downstream pathways and that TLR3 signals predominantly through a MyD88-independent pathway [17,23], although the initial study suggests that MyD88 contributes to TLR3-cytokine production [19]. Several other studies even suggest that MyD88 negatively regulates TLR3-dependent cytokine production and inflammation [25,26]. To further investigate whether MyD88 contributes to TLR3 signaling, we re-examined NF-κB activation and cytokine production in response to the TLR3 agonist poly(I:C) using mouse bone marrow-derived macrophages (BMDMs). Mouse BMDMs were isolated from WT, TRIF^-/-^, and MyD88^-/-^ mice as described previously [5,8,9]. Depletion of the individual gene was verified by Western blot (Figure 1A,B). NF-κB activation was evaluated by assessing p65 and IκBα phosphorylation, as well as IκBα degradation. Poly(I:C) triggered significant phosphorylation of p65 and IκBα in WT BMDMs (Figure 1C–G). As expected, phosphorylation of p65 and IκBα was diminished in TRIF^-/-^ cells (Figure 1C–G), consistent with the established role of TRIF in TLR3 signaling. Unexpectedly, p65 and IκBα phosphorylation were also reduced in MyD88^-/-^ cells, although to a lesser extent (Figure 1C–G). Accordingly, IκBα was degraded upon poly(I:C) stimulation at 60 and 120 min in WT BMDMs, but this degradation was prevented in TRIF^-/-^ cells. IκBα degradation was also reduced in MyD88^-/-^ cells at 120 min (Figure 1C,F). The reduction in NF-κB activation observed in MyD88^-/-^ cells in response to poly(I:C) was not due to altered expression of TLR3 or TRIF (Figure 1A,B). Taken together, these data suggest that, in addition to TRIF, MyD88 also contributes to NF-κB activation downstream of TLR3.

### 3.2. MyD88 Contributes to the Transcription of Cytokines

**Figure 2:**
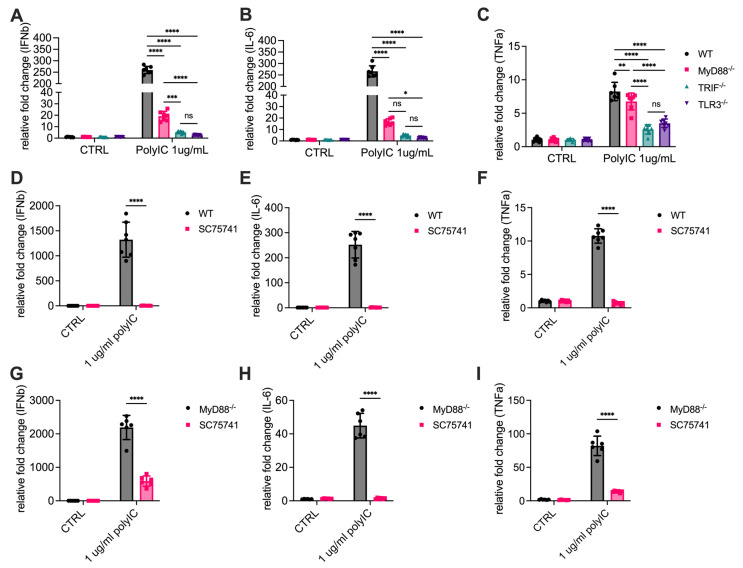
MyD88 contributes to NF-κB-dependent cytokine transcription in response to poly(I:C) stimulation. (**A**–**C**) Primary macrophages from WT, MyD88^-/-^, TRIF^-/-^, and TLR3^-/-^ mice were stimulated with 1 µg/mL Poly(I:C) for 2 h. (**D**–**F**) WT macrophages or (**G**–**I**) MyD88^-/-^ macrophages were pretreated with the NF-κB inhibitor SC75741 for 1 h before stimulation with 1 µg/mL Poly(I:C) for 2 h. Total RNA was extracted, and mRNA levels of cytokines were analyzed by quantitative RT-PCR (qRT-PCR). Relative expression levels of (**A**,**D**,**G**) IFN-β, (**B**,**E**,**H**) IL-6, and (**C**,**F**,**I**) TNF-α were normalized to a reference gene and presented as fold changes compared to untreated controls (CTRL). Data are presented as mean ± SD (N = 7 for (**A**–**F**) or 6 for (**G**–**I**), biological replicates). Statistical analysis for comparisons among multiple groups was performed using two-way ANOVA followed by Sidak’s multiple comparisons testing. ns, not significant, * *p* < 0.05, ** *p* < 0.01, *** *p* < 0.005, **** *p* < 0.0001.

Activation of NF-κB is a critical driver of gene transcription, particularly for pro-inflammatory cytokines and interferons, which play essential roles in innate immune responses. To investigate whether MyD88 contributes to TLR3-induced expression of pro-inflammatory cytokines, we measured mRNA expression levels of key cytokines IL-6, TNF-α, and IFN-β in response to poly(I:C) stimulation. Mouse BMDMs isolated from WT, TLR3^-/-^, TRIF^-/-^, and MyD88^-/-^ mice were treated with poly(I:C), and total RNA was extracted after 2 h for quantitative PCR (qPCR) analysis. In WT cells, poly(I:C) treatment resulted in a robust increase in IL-6, TNF-α, and IFN-β mRNA levels, consistent with an active transcriptional response to TLR3 signaling. TLR3^-/-^ cells exhibited minimal induction of these cytokines, indicating that poly(I:C)-induced cytokine production is mediated through TLR3 (Figure 2A–C). As expected, TRIF^-/-^ cells also showed minimal induction of these cytokines, confirming the essential roles of TRIF in TLR3 signaling. Importantly, MyD88^-/-^ cells also showed significantly reduced mRNA levels of IL-6 and IFN-β, but not TNF-α, compared to WT cells (Figure 2A–C, Supplementary Figure S3). These results demonstrate that MyD88 is essential for optimal transcriptional activation of these cytokines downstream of TLR3.

To confirm that this transcriptional activation is mediated by NF-κB, pretreatment with an NF-κB inhibitor SC75741 completely abolished the induction of IL-6, TNF-α, and IFN-β mRNA, consistent with their dependence on NF-κB signaling (Figure 2D–F). We also performed the experiments in MyD88-deficient cells (Figure 2G–I). It showed that SC75741 also abrogated the induction of IL-6, TNF-α, and IFN-β at the mRNA in MyD88-deficient cells, indicating that the MyD88-dependent effects are also NF-κB–dependent. These results suggest that the impaired cytokine transcription observed in MyD88^-/-^ cells is due to defects in NF-κB activation, highlighting the critical role of MyD88 in regulating TLR3-mediated gene expression.

### 3.3. Cytokine Release Is Significantly Reduced in MyD88^-/-^ Cells

**Figure 3:**
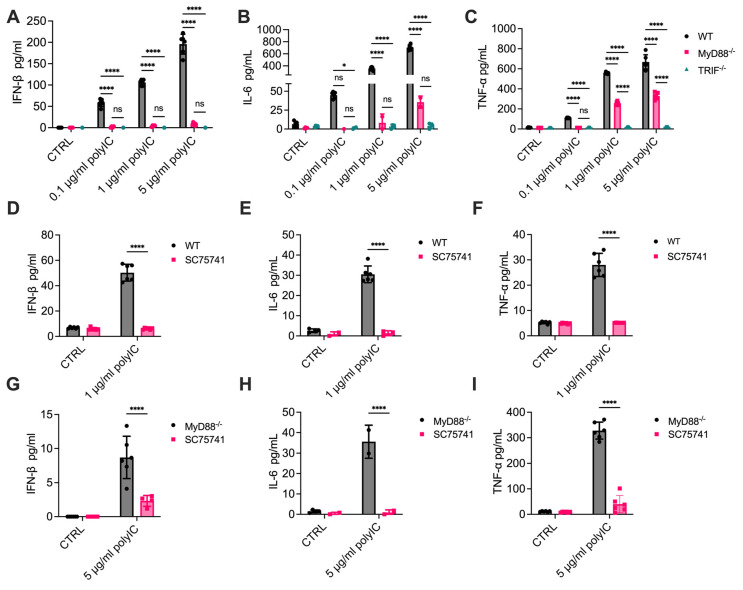
MyD88 contributes to NF-κB-dependent cytokine production in response to poly(I:C) stimulation. (**A**–**C**) Primary macrophages from WT, MyD88^-/-^, and TRIF^-/-^ mice were stimulated with increasing concentrations of poly(I:C) (0.1, 1, or 5 µg/mL) for 6 h. (**D**–**F**) WT macrophages and (**G**–**I**) MyD88^-/-^ macrophages were pretreated with the NF-κB inhibitor SC75741 for 1 h before stimulation with 1 or 5 µg/mL Poly(I:C) for 6 h. Cytokine levels in the supernatants were measured by ELISA: (**D**,**G**) IFN-β, (**E**,**H**) IL-6, and (**F**,**I**) TNF-α. Data are presented as mean ± SD (N = 6, biological replicates). Statistical analysis for comparisons among multiple groups was performed using two-way ANOVA followed by Sidak’s multiple comparisons testing. ns, not significant, * *p* < 0.05, **** *p* < 0.0001.

Next, we measured cytokine levels in the supernatants of cells stimulated with increasing amounts of poly(I:C) for 6 h by ELISA assays to further determine the role of MyD88 in TLR3-dependent pro-inflammatory cytokine production. In WT cells, poly(I:C) robustly induced the secretion of IL-6, TNF-α, and IFN-β, indicating effective activation of TLR3. As expected, TRIF^-/-^ cells showed significantly reduced cytokine secretion, consistent with its established role as a key adaptor in TLR3 signaling. Poly(I:C) induced IL-6, TNF-α, and IFN-β production was also significantly reduced in MyD88^-/-^ cells, although the reduction was less severe than that observed in TRIF^-/-^ cells (Figure 3A–C, Supplementary Figure S4). To further confirm that these cytokines are downstream products of the NF-κB signaling pathway, WT cells were pretreated with the specific NF-κB inhibitor SC75741 for 1 h prior to poly(I:C) stimulation. Inhibition of NF-κB almost completely abolished the secretion of IL-6, TNF-α, and IFN-β, confirming their dependence on NF-κB activity (Figure 3D–F). Similar results were also shown in MyD88-deficient cells (Figure 3G–I). These findings suggest that the observed reduction in cytokine secretion in MyD88^-/-^ cells is primarily due to impaired NF-κB activation.

In addition to inducing cytokine release, poly(I:C) stimulation caused a slight but significant increase in cell death in WT cells compared to untreated controls, as measured by LDH assay. Poly(I:C)-induced cell death was markedly reduced in MyD88^-/-^ cells and completely abolished in TLR3^-/-^ and TRIF^-/-^ cells (Supplementary Figure S1). These findings indicate that cell death in response to TLR3 stimulation is closely linked to NF-κB signaling and requires the presence of both TRIF and MyD88. Overall, these results demonstrate that MyD88 is essential for optimal cytokine production downstream of TLR3. The residual cytokine release observed in MyD88^-/-^ cells may suggest partial compensation by TRIF or other pathways. Together, these findings provide strong evidence for a previously unrecognized role of MyD88 in regulating TLR3-dependent cytokine secretion.

### 3.4. Attenuated Cytokine Responses in MyD88^-/-^ Mice

**Figure 4:**
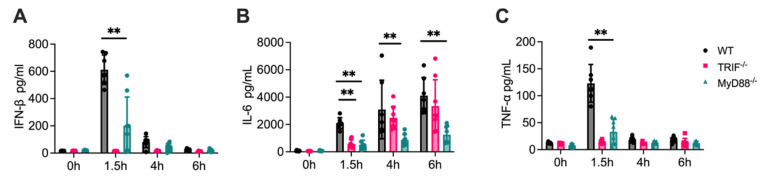
Poly(I:C)-induced cytokine production in vivo involves both TRIF and MyD88 signaling. Mice were injected intraperitoneally with 50 µg Poly(I:C) per mouse. At the indicated time points (0 h, 1.5 h, 4 h, and 6 h), blood samples were collected via retro-orbital bleeding, and cytokine levels in the serum were quantified by ELISA. (**A**) IFN-β levels, (**B**) IL-6 levels, and (**C**) TNF-α levels are shown for WT, TRIF^-/-^, and MyD88^-/-^ mice. Data are presented as mean ± SD (N = 8, biological replicates), with significant differences indicated as ** *p* < 0.01.

To investigate the role of MyD88 in TLR3-mediated systemic cytokine response in vivo, WT, TRIF^-/-^, and MyD88^-/-^ mice were injected intraperitoneally with poly(I:C), and plasma levels of IL-6, TNF-α, and IFN-β were measured at various time points post-injection. In WT mice, poly(I:C) induced distinct dynamic changes in plasma cytokine levels. TNF-α and IFN-β reached peak concentrations at 1.5 h and then rapidly declined, returning to near-baseline levels by 4 and 6 h. In contrast, IL-6 exhibited a continuous increase over time, reaching its highest levels at 6 h (Figure 4A–C).

In TRIF^-/-^ mice, plasma levels of TNF-α and IFN-β remained at baseline across all time points, indicating that TRIF is essential for the production of these cytokines in response to poly(I:C). Similarly, IL-6 levels in TRIF^-/-^ mice were significantly lower than those in WT mice at 1.5 h, suggesting that TRIF contributes to the early induction of IL-6. However, by 4 and 6 h, IL-6 levels in TRIF^-/-^ mice were comparable to those in WT mice, indicating that later-phase IL-6 production is less dependent on TRIF and may involve alternative pathways.

In MyD88^-/-^ mice, serum levels of all three cytokines were significantly reduced compared to WT mice, highlighting the critical role of MyD88 in amplifying and sustaining TLR3-mediated responses. TNF-α and IFN-β levels in MyD88^-/-^ mice were markedly lower than WT levels at 1.5 h and remained near baseline at 4 and 6 h. For IL-6, MyD88^-/-^ mice exhibited consistently lower levels than WT mice across all time points. Notably, even at 6 h, when IL-6 levels in WT mice peaked, IL-6 concentrations in MyD88^-/-^ mice were significantly reduced. This suggests that MyD88 is crucial for both early- and late-phase IL-6 production.

Together, these results reveal the distinct dependencies of TLR3-mediated systemic cytokine responses on TRIF and MyD88.

### 3.5. MyD88 Participation in TLR3 Signaling Extends Beyond Poly(I:C)

**Figure 5:**
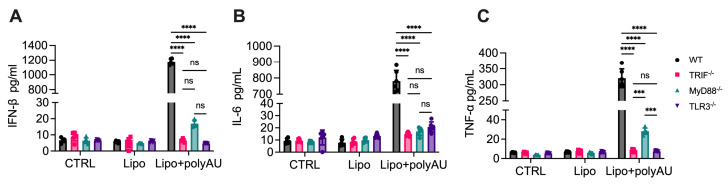
MyD88 contributes to cytokine production in response to poly(A:U). (**A**–**C**) Primary macrophages from WT, MyD88^-/-^, TRIF^-/-^, and TLR3^-/-^ mice were transfected with 250 µg/mL poly(A:U) using Lipofectamine 3000 for 24 h. Cytokine levels in the supernatants were measured by ELISA. (**A**) IFN-β, (**B**) TNF-α, and (**C**) IL-6 levels are shown. Data are presented as mean ± SD (N = 6, biological replicates). Statistical analysis for comparisons among multiple groups was performed using two-way ANOVA followed by Sidak’s multiple comparisons testing. ns, not significant, *** *p* < 0.005, **** *p* < 0.0001.

TLR3 plays a critical role in antiviral immunity by recognizing double-stranded RNA (dsRNA), a molecular signature of many viruses. Poly(I:C) is a synthetic dsRNA analog and a well-established TLR3 agonist. In addition to poly(I:C), other synthetic dsRNA analogs, such as RGC100, ARNAX, PolyA, TL-532, and poly(A:U) [27,28], have been developed as TLR3 agonists, primarily for use as vaccine adjuvants. To further evaluate the contribution of MyD88 in TLR3 signaling, we tested its role of MyD88 in poly(A:U)-induced cytokine production. Unlike poly(I:C), which is commonly used as a TLR3 agonist, poly(A:U) typically consists of 200–4000 base pairs, resulting in a higher molecular weight that limits its ability to be internalized into macrophages via endocytosis [29]. To overcome this limitation of poly(A:U), we utilized a transfection reagent Lipofectamine 3000 to deliver poly(A:U) into macrophages. This approach effectively bypassed size constraints and allowed intracellular delivery of poly(A:U). Under these conditions, poly(A:U) stimulation triggered cytokine release similar to that induced by poly(I:C). We observed that this cytokine response was also MyD88-dependent, as cytokine production and NF-κB activation were significantly impaired in MyD88^-/-^ cells, whereas TRIF^-/-^ cells completely lost responsiveness (Figure 5A–C). These data confirmed the role of MyD88 in TLR3 signaling, as intracellularly delivered poly(A:U) induces TLR3-mediated cytokine production and NF-κB activation in a MyD88-dependent manner, similar to poly(I:C).

### 3.6. Poly(I:C) Stimulation Enhances the Direct Association Between TLR3 and MyD88

**Figure 6:**
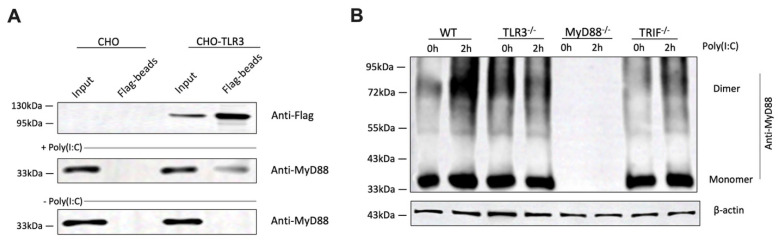
Poly(I:C) stimulation induces TLR3 and MyD88 interaction and MyD88 oligomerization. (**A**) CHO and CHO cells transfected with FLAG-tagged TLR3 (CHO-TLR3) were stimulated with 1 µg/mL poly(I:C) for 2 h. Cell lysates were subjected to co-immunoprecipitation using anti-FLAG beads, followed by immunoblotting with anti-FLAG and anti-MyD88 antibodies to detect the interaction between TLR3 and MyD88. (**B**) BMDMs from WT, MyD88^-/-^, and TLR3^-/-^ mice were stimulated with 1 µg/mL poly(I:C) for the indicated times. Cell lysates were treated with DSS for 2 h, then analyzed by SDS-PAGE and immunoblotted with anti-MyD88 antibody to assess oligomerization status. β-actin was used as a loading control.

To explore the underlying mechanism by which MyD88 contributes to TLR3 signaling, we investigate a potential interaction between MyD88 and TLR3. Initial attempts using commercial anti-TLR3 antibodies failed to efficiently pull down TLR3 in cell lysates. Therefore, we constructed a plasmid to express C-terminal FLAG-tagged TLR3 (TLR3-FLAG) and transfected it into CHO cells. CHO cells naturally express high levels of MyD88, making them a suitable model for studying the interaction between these two proteins. Forty-eight hours after transfection, we performed co-immunoprecipitation (co-IP) to examine whether there was a direct interaction between TLR3 and MyD88. CHO cells expressing TLR3-FLAG or WT CHO cells were incubated with or without poly(I:C) for 2 h and then lysed by a lysis buffer. Anti-FLAG beads successfully pulled down MyD88 from cells expressing TLR3-FLAG, but only upon poly(I:C) stimulation (Figure 6A). These data indicate a potential interaction between MyD88 and TLR3 following TLR3 activation (Figure 6A).

Previous studies have shown that MyD88 can form dimers to participate in the TLR4 signaling pathway [30,31]. To investigate whether MyD88 is also involved in TLR3-mediated signaling, we examined its oligomerization status following poly(I:C) stimulation. Indeed, poly(I:C) stimulation promoted MyD88 oligomerization (Figure 6B). However, in TLR3^-/-^ cells, MyD88 oligomerization was not observed, suggesting that this process is dependent on the presence of TLR3 (Figure 6B). As a positive control, MyD88 dimers were also detected when cells were primed with LPS (Supplementary Figure S2). MyD88^-/-^ cells were used as a negative control to confirm the specificity of the anti-MyD88 antibody. Interestingly, poly(I:C)-induced MyD88 oligomerization was abolished in the TRIF deficient cells, suggesting that TLR3-dependent MyD88 activation occurs downstream from TRIF signaling. We quantified MyD88 oligomerization at 0 and 2 h after stimulation (Supplementary Figure S5). The analysis demonstrates that poly(I:C)–induced MyD88 oligomerization is substantially diminished in TRIF-deficient cells, although the 2 h-band is slightly darker than the 0 h-band.

## 4. Discussion

TLR3 plays a crucial role in the innate immune response by recognizing viral infection markers, dsRNAs. Upon activation, TLR3 promotes the production of type I interferons (IFNs) and other pro-inflammatory cytokines, providing a critical defense against viral pathogens. This signaling pathway also contributes to the activation of adaptive immunity, making TLR3 a promising target for interventions such as antiviral drugs and vaccine adjuvants, especially for reducing the length of stay and hospital resource burden [32,33,34,35,36]. TLR3 activation can enhance complement activity, boosting immune responses and vaccine efficacy [37]; however, excessive activation may cause tissue damage, as seen in complement-driven endothelial injury in Transplantation-associated thrombotic microangiopathy (TA-TMA) [38,39]. Therefore, understanding the molecular mechanisms underlying TLR3-mediated cytokine production may offer novel insights into antiviral immunity. Traditionally, TLR3 has been considered unique among TLRs because it signals exclusively through TRIF, bypassing the MyD88-dependent pathway utilized by most other TLRs. Furthermore, previous studies have suggested that MyD88 may serve as a negative regulator or TLR3 signaling, limiting IRF3 activation and cytokine production [25,26]. However, our data demonstrate that MyD88 contributes to TLR3-mediated NF-κB activation and cytokine production, thereby challenging the current paradigm that TLR3 signaling is entirely MyD88-independent.

Poly(I:C), a classic TLR3 agonist, has been widely reported to induce apoptosis in tumor cells through the TLR3 signaling pathway [40,41]. Compared to wild-type cells, poly(I:C) stimulation resulted in reduced phosphorylation of p65 and IκBα in MyD88 deficient macrophages. This impaired NF-κB activation was not limited to poly(I:C) stimulation; a similar reduction in NF-κB activation was also observed with another TLR3 agonist, poly(A:U), indicating that MyD88’s involvement in TLR3 signaling is not agonist-specific. These findings highlight a previously underappreciated role of MyD88 in regulating TLR3-dependent NF-κB activation. Consistent with the role of MyD88 in NF-κB activation, MyD88 deficient macrophages exhibited significantly reduced mRNA levels and secretion of cytokines, including IL-6, TNF-α, and IFN-β, following stimulation with the TLR3 agonist poly(I:C). These data suggest that MyD88 is essential for the optimal transcriptional activation of cytokine genes downstream of TLR3. Consistent with the in vitro findings, poly(I:C) induced less cytokine production in MyD88 deficient mice. These data indicate that MyD88 is not merely a passive bystander in TLR3 signaling but actively promotes cytokine production. Incubation of macrophages with poly(I:C) significantly induced macrophage death, which was also TRIF-dependent, as TRIF deficiency completely blocked poly(I:C)-induced macrophage death. Cell death was significantly attenuated in the BMDMs from MyD88^-/-^ mice, suggesting that MyD88 also plays an important role in regulating TLR3-mediated macrophage death. IFN-β release is known to be regulated by the TRIF–TBK1–IRF3 signaling pathway. In our study, we examined TBK1 phosphorylation following poly(I:C) stimulation (Supplementary Figure S6). In WT BMDMs, TBK1 phosphorylation was strongly induced between 30 and 90 min. In contrast, MyD88^-/-^ cells showed a clear reduction in phosphorylation at 90 min, while TRIF^-/-^ cells displayed persistently low levels throughout the entire time course. These findings highlight the essential role of TRIF in mediating TBK1 activation, with MyD88 playing a potential modulatory role in sustaining the response.

While it has been well accepted that TLR3-induced IFN-β production relies entirely on the TRIF pathway and is independent of MyD88, the role of MyD88 in TLR3-induced NF-κB activation remains controversial. TLR3 was first identified in 2001 as a receptor that recognizes double-stranded RNA (dsRNA) and its synthetic analog poly(I:C) [19]. Consistent with our findings, Alexopoulou et al. reported that, compared to wild-type cells, the induction of IL-12, IL-6, and NO_2_ by poly(I:C) was impaired in MyD88-deficient dendritic cells and macrophages, suggesting a role for MyD88 in TLR3 signaling [19]. However, this concept is later abandoned after two subsequent studies showed that poly(I:C)-induced cytokine production is entirely dependent on TRIF signaling [17,23]. Yamamoto et al. reported that MyD88-deficient cells still responded normally to poly(I:C) and disrupting TRIF abolished TLR3-mediated IFN-β induction and IRF3 activation [17]. These results are consistent with the findings from a mouse model carrying a distal frameshift mutant in TRIF, known as Lps2 [23]. TRIF^Lps2^ mutant macrophages failed to produce IFN-α/β in response to poly(I:C) stimulation. Both studies showed that poly(I:C)-induced NF-κB activation and TNF-α production are dramatically diminished in macrophages, which is contradictory to our findings. However, in both studies, peritoneal macrophages isolated from thioglycolate-treated mice were used. It is known that the injection of a medium amount of thioglycollate into the peritoneal cavity triggers the recruitment and activation of macrophages. Therefore, the macrophages they used may be desensitized by thioglycollate pre-treatment and rendered less responsive to subsequent stimuli. In contrast, we used mouse BMDMs, which are maintained under more resting conditions and are likely to be more responsive to poly(I:C) stimulation. In supporting this hypothesis, Siednienko et al. reported that while MyD88 negatively regulates TLR3-induced IFN-β and RANTES production, poly(I:C)-induced and TNF-α production is actually reduced in mouse BMDMs [26].

Our findings further highlight the critical role of MyD88 in NF-κB activation downstream of TLR3. Importantly, we report for the first time that MyD88 undergoes oligomerization during TLR3 activation, which is abolished in TLR3^-/-^ cells. Our co-immunoprecipitation experiments revealed an interaction between MyD88 and TLR3 following TLR3 activation. These findings suggest that MyD88 may form a signaling complex with TLR3, potentially facilitating or stabilizing downstream signaling events, at least in macrophages. This observation opens up new avenues for understanding the molecular mechanisms by which MyD88 participates in TLR3 signaling. It has been previously reported that TLR3 physically associates with TRIF [18]. Our data do not exclude the possibility that MyD88 may associate indirectly with TLR3 through TRIF, and that MyD88 activation via TLR3 occurs downstream of TRIF. This hypothesis may explain why TLR3 signaling is entirely dependent on TRIF yet also involves the MyD88 pathway. In support of this hypothesis, we show that poly(I:C)-induced MyD88 oligomerization was abolished not only by TLR3 deficiency but also by TRIF deficiency. As previously reported by Teixeira et al. [42], stimulation of TLR3 by poly(I:C) can induce the release of damage-associated molecular patterns (DAMPs) like HMGB1. We measured HMGB1 release after poly(I:C) stimulation (Supplementary Figure S7). Poly(I:C) induced HMGB1 release from BMDMs, and this response was abolished in TRIF- or MyD88-deficient cells. These findings indicate that HMGB1 release is downstream, rather than upstream, of MyD88 signaling in our system. Nevertheless, we cannot exclude the possibility that TLR3-dependent MyD88 activation occurs via a positive-feedback process secondary to initial RLR3 signaling, rather than through direct coupling of TLR3 to MyD88.

The ligands of TLR7 are mainly single-stranded RNA (ssRNA) and small-molecule compounds such as Imidazoquinoline derivatives (e.g., R848 and R837) [43]. A study by Takahiro et al. demonstrated that poly(A:U) can function as an immune adjuvant in vivo through both TLR3 and TLR7 [44]. Specifically, poly(A:U) was shown to induce the production of IFN-α and IL-12 in mouse bone marrow-derived dendritic cells via a TLR7-dependent pathway, whereas in conventional dendritic cells with high CD24 expression (CD24^high^ cDCs), IL-12 secretion was mainly dependent on TLR3. In addition, in vivo injection of OVA together with poly(A:U) was able to elicit TLR3- and TLR7-dependent expansion of CD8^+^ T cells. In our study, we found that poly(A:U)-induced cytokine production in BMDMs was almost completely abolished in TLR3-deficient cells, suggesting that these responses are mainly mediated through a TLR3-dependent pathway. This discrepancy may be related to cell type specificity. Future studies should further validate this by using TLR7-deficient cells to clarify the role of TLR7 in poly(A:U)-induced immune responses.

In summary, our study suggests that TRIF plays an essential role in TLR3 signaling, while MyD88 contributes to this role. Specifically, we observed that TRIF^-/-^ cells exhibited nearly identical suppression of cytokine secretion and NF-κB activation as TLR3^-/-^ cells, indicating that TLR3 signaling is highly dependent on TRIF. In contrast, the absence of MyD88 resulted in the partial, but significant, inhibition of TLR3 signaling. Based on these findings, we hypothesize that MyD88 contributes to the assembly or stability of the TLR3-TRIF signaling complex and provides structural support for the coordinated activation of NF-κB and IRF3, rather than acting as an indispensable adaptor protein. Future studies should focus on elucidating the interaction between MyD88 and TRIF. Additionally, further exploration is needed to determine the universality of this interaction across different cell types and its functional significance in antiviral immunity and inflammatory diseases. These investigations will help deepen our understanding of the complexity of the TLR3 signaling network and may provide novel strategies for the targeted modulation of TLR3 signaling.

## Figures and Tables

**Table 1:** Antibody Information.

Antibody	Clonality	Clone ID	Host Species	Target Epitope	Vendor	Catalog #	Lot #
TLR3	Monoclonal	40C1285.6	mouse	Amino acids 55–85	Novus Biologicals	NBP2-24875	C-5
MyD88	Monoclonal	D80F5	rabbit	Cys233	CST	4283	/
TRIF	Polyclonal	/	rabbit	amino acids 100–180	Novus Biologicals	NB120-13810SS	B-4
p65	Monoclonal	D14E12	rabbit	Glu498	CST	8242	/
p-p65	Polyclonal	/	rabbit	Ser536	CST	3031	/
IκB	Monoclonal	L35A5	mouse	Amino-terminal	CST	4814	/
p-IκB	Monoclonal	14D4	rabbit	Ser32	CST	2859	/
Flag	Monoclonal	M2	Mouse	FLAG	Sigma-Aldrich	F1804	/
β-actin	Monoclonal	13E5	rabbit	amino-terminal	CST	4970	/

## Data Availability

All research data has been included in the manuscript.

## Supplementary Materials

The following supporting information can be downloaded at https://www.mdpi.com/article/10.3390/cells14191507/s1: Figure S1: poly(I:C)-induced cell death was dependent on MyD88; Figure S2: LPS induced MyD88 oligomerization; Figure S3: Comparative effect sizes (95% CI) across multiple groups under the experimental settings (Figure 2A–C); Figure S4: Comparative effect sizes (95% CI) across multiple groups under the experimental settings (Figure 3A–C); Figure S5: Quantitative analysis of MyD88 oligomerization (shown in Figure 6B); Figure S6: TRIF-TBK1-IRF3 activation in response to poly(I:C); Figure S7: TRIF-TBK1-IRF3 activation in response to poly(I:C).

## Abbreviations

The following abbreviations are used in this manuscript:

TLR3	Toll-like receptor 3
TRIF	TIR-domain-containing adapter-inducing interferon-β
MyD88	myeloid differentiation primary response 88
poly(I:C)	polyinosinic-polycytidylic acid
poly(A:U)	polyadenylic–polyuridylic acid
BMDMs	bone marrow-derived macrophages
TLRs	Toll-like receptors
PRRs	pattern recognition receptors
PAMPs	pathogen-associated molecular patterns
LPSs	lipopolysaccharides
IFN-I	type I interferons
dsRNA	double-stranded RNA
NF-κB	nuclear factor kappa-light-chain-enhancer of activated B cells
IκB	Inhibitor of kappa B
MAPKs	mitogen-activated protein kinases
TNF-α	tumor necrosis factor-alpha
IRF3	interferon regulatory factor 3
RT-PCR	Reverse Transcription Polymerase Chain Reaction
qPCR	quantitative PCR
WT	Wild type
IL	interleukin
LDH	lactate dehydrogenase
DSS	disuccinimidyl suberate
