# Supplementary material for: MyD88 Contributes to TLR3-Mediated NF-κB Activation and Cytokine Production in Macrophages
Source: Cells. 2025 Sep 27;14(19):1507. doi: 10.3390/cells14191507 (PMC12523770; doi:10.3390/cells14191507)
Supplement: Supplementary file 1 [file cells-14-01507-s001.zip › cells-3827915-supplementary.pdf]

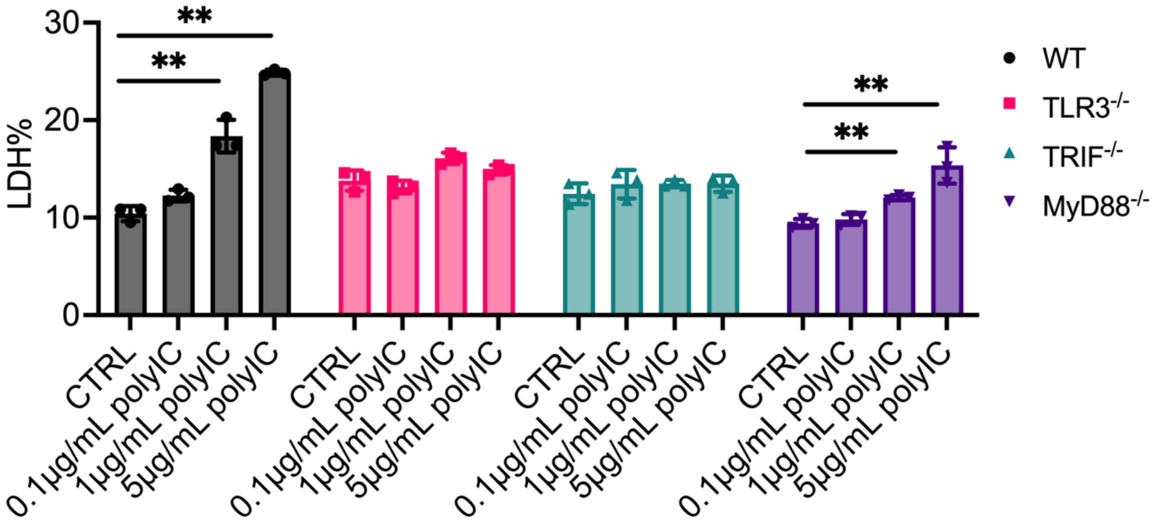

Figure. S1 poly(I:C) induced cell death was dependent to MyD88. BMDMs from WT, TLR3$^{-/-}$, TRIF$^{-/-}$, and MyD88$^{-/-}$ mice were stimulated with increasing concentrations of Poly(I:C) (0.1 μg/mL, 1 μg/mL, and 5 μg/mL) for 6 hours. Cell death was quantified using an LDH release assay and is presented as the percentage of total LDH release. Data are presented as mean ± SD (N = 3). $^{**}p < 0.01$.

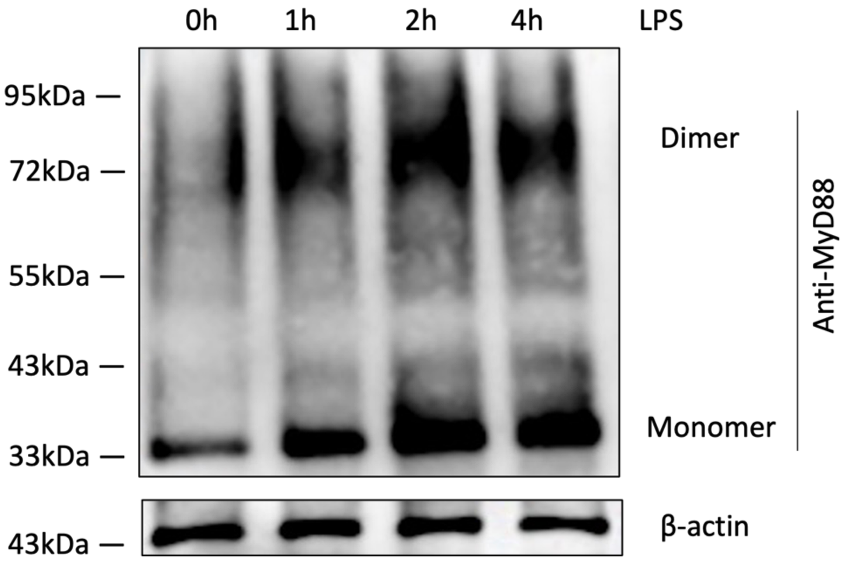

Figure. S2 LPS induced MyD88 oligomerization. BMDMs from WT mice were stimulated with 100 ng/mL LPS for the indicated hours. Cell lysates were treated with DSS for 2h, then analyzed by SDS-PAGE and immunoblotted with anti-MyD88 antibody to assess oligomerization status. β-actin was used as a loading control.

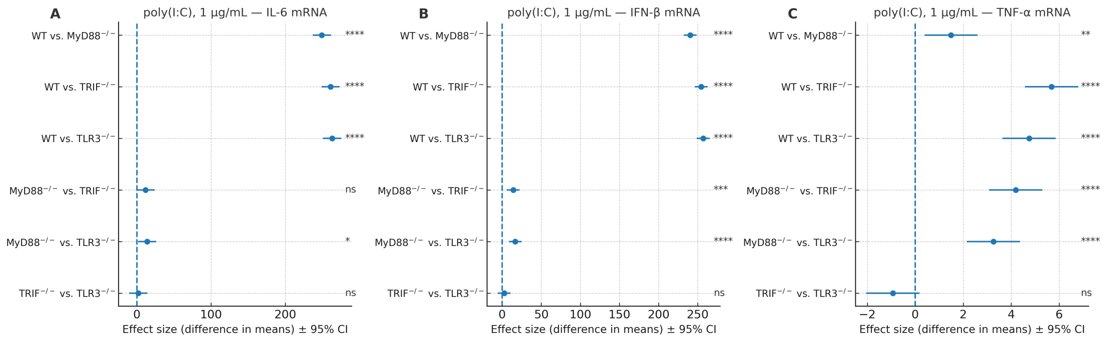

Figure. S3 Comparative effect sizes (95% CI) across multiple groups under the experimental settings (Figure 2A–C). Primary macrophages from WT, MyD88$^{-/-}$, TRIF$^{-/-}$, and TLR3$^{-/-}$ mice were stimulated with 1 µg/mL Poly(I:C) for 2 hours..

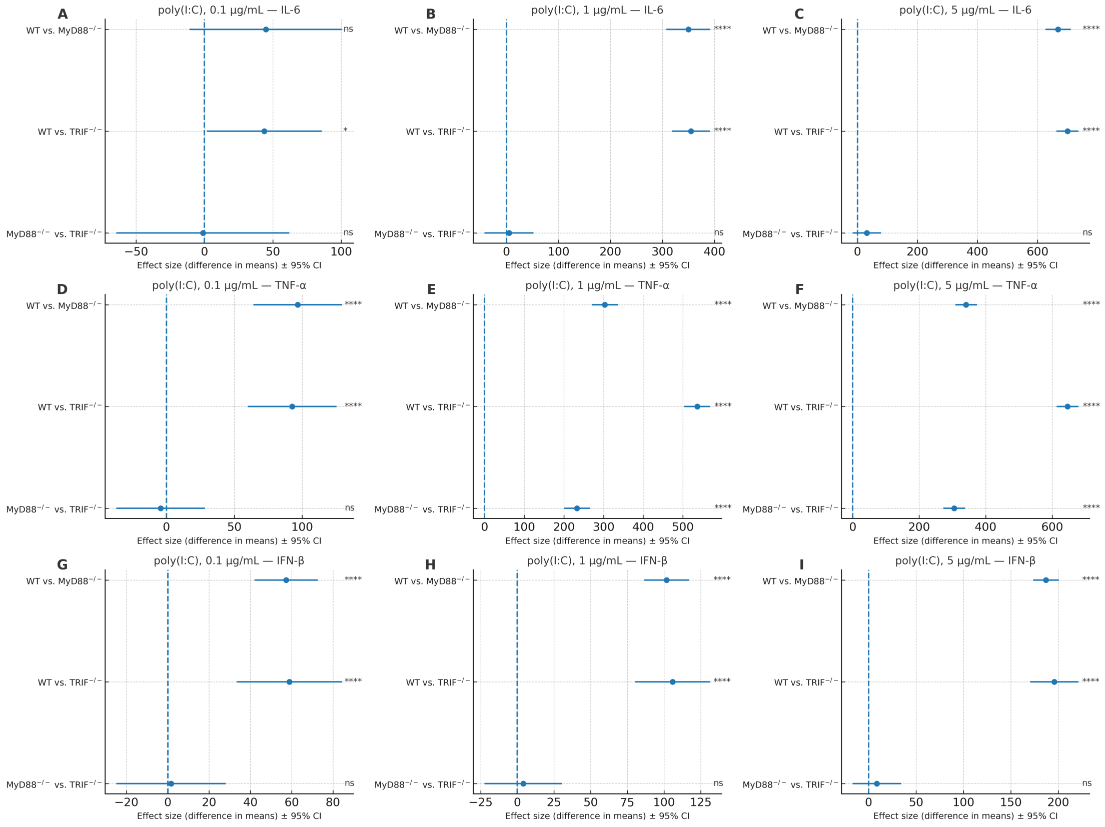

Figure. S4 Comparative effect sizes (95% CI) across multiple groups under the experimental settings (Figure 3A–C). Primary macrophages from WT, MyD88⁻/⁻, and TRIF⁻/⁻ mice were stimulated with increasing concentrations of poly(I:C) (0.1, 1, or 5 μg/mL) for 6 hours.

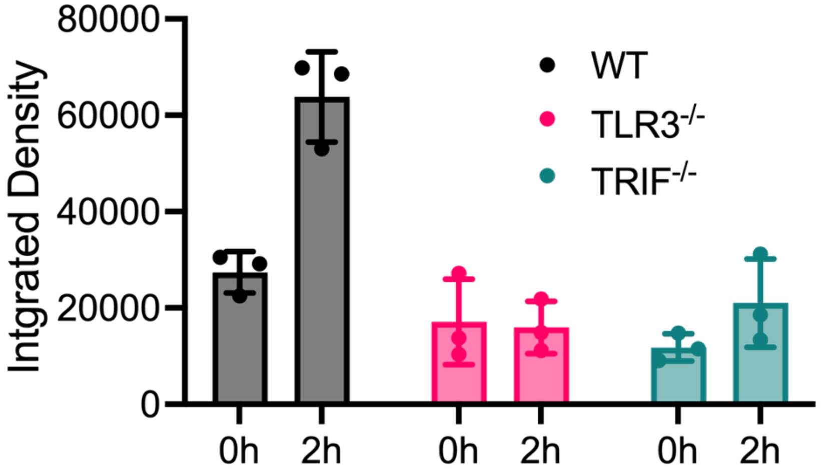

Figure. S5 Quantitative analysis of MyD88 oligomerization (shown in Figure 6B). Primary macrophages from WT, MyD88$^{-/-}$, and TRIF$^{-/-}$ mice were stimulated with 1 μg/mL concentrations of poly(I:C) for 2 hours.

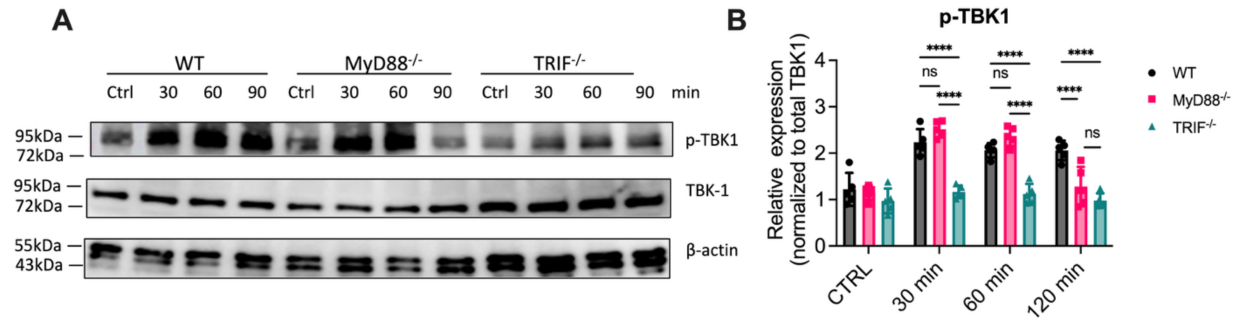

Figure. S6 TRIF-TBK1-IRF3 activation in response to poly(I:C). (A) Cells were stimulated with 1 μg/mL poly(I:C), and total protein lysates were collected at the indicated time points (0, 30, 60, and 90 minutes). Western blot analysis was performed to detect total TBK1 and phosphorylated TBK1 (p-TBK1), with β-actin used as a loading control. (B) Quantification of protein band intensities from (A) was performed using ImageJ software. Relative intensities of p-TBK1 was normalized to total TBK1 and presented as fold changes compared to controls (CTRL). Data are presented as mean ± SD (N=5, biological replicates). Statistical analysis for comparisons among multiple groups was performed using two-way ANOVA followed by Sidak's multiple comparisons testing. *p < 0.05, **p < 0.01, ***p < 0.005, ****p < 0.0001.

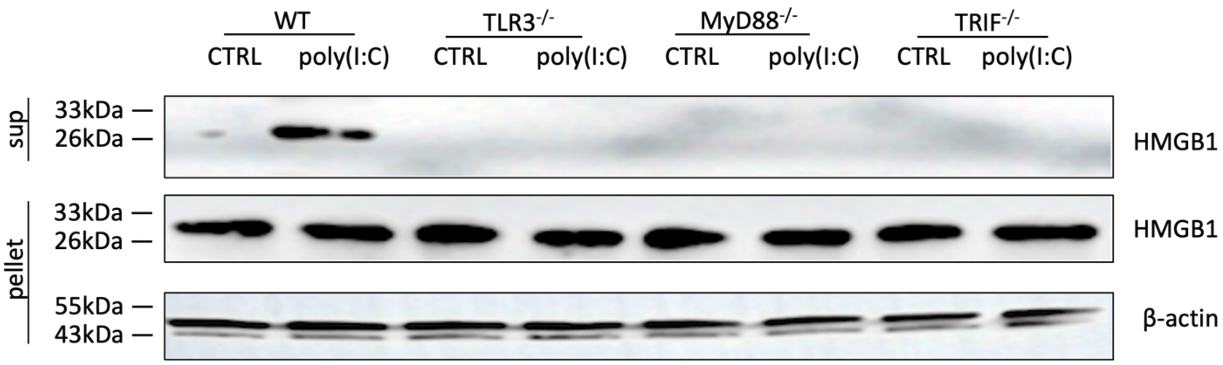

Figure. S7 TRIF-TBK1-IRF3 activation in response to poly(I:C). Cells were stimulated with 1 μg/mL poly(I:C) for 6h. Western blot analysis was performed to detect HMGB1 in both medium (sup) and cell lysate (pellet) , with β-actin used as a loading control.
